# Repurposing Drugs in Oncology (ReDO)—diclofenac as an anti-cancer agent

**DOI:** 10.3332/ecancer.2016.610

**Published:** 2016-01-11

**Authors:** Pan Pantziarka, Vidula Sukhatme, Gauthier Bouche, Lydie Meheus, Vikas P Sukhatme

**Affiliations:** 1 Anticancer Fund, Brussels, 1853 Strombeek-Bever, Belgium; 2 The George Pantziarka TP53 Trust, London, UK; 3 GlobalCures, Inc; Newton MA 02459, USA; 4 Beth Israel Deaconess Medical Center and Harvard Medical School, Boston, MA 02215, USA

**Keywords:** drug repurposing, diclofenac, NSAID, perioperative intervention, ReDO project

## Abstract

Diclofenac (DCF) is a well-known and widely used non-steroidal anti-inflammatory drug (NSAID), with a range of actions which are of interest in an oncological context. While there has long been an interest in the use of NSAIDs in chemoprevention, there is now emerging evidence that such drugs may have activity in a treatment setting. DCF, which is a potent inhibitor of COX-2 and prostaglandin E2 synthesis, displays a range of effects on the immune system, the angiogenic cascade, chemo- and radio-sensitivity and tumour metabolism. Both pre-clinical and clinical evidence of these effects, in multiple cancer types, is assessed and summarised and relevant mechanisms of action outlined. Based on this evidence the case is made for further clinical investigation of the anticancer effects of DCF, particularly in combination with other agents - with a range of possible multi-drug and multi-modality combinations outlined in the supplementary materials accompanying the main paper.

## Introduction

Diclofenac (DCF) is a commonly used non-steroidal anti-inflammatory drug (NSAID) used in the treatment of pain in rheumatoid arthritis and other musculoskeletal conditions, migraine, fever, acute gout and post-operative pain. First developed by Ciba-Geigy (later merging with Sandoz to become Novartis), the drug is now available globally as a generic medication. DCF is also commonly available as a gel for topical application for localised pain or for the treatment of actinic keratosis. In some countries low-dose formulations of oral DCF (typically 25 mg tablets in small pack sizes of 12 – 18 tablets) are available over-the-counter (OTC) as a general purpose analgesic or anti-pyretic. It is also generally available OTC in the gel format. Common trade names include Voltaren, Voltarol, Cataflam, Cambia, Zipsor and Zorvolex.

DCF has an established role in oncological practice in the treatment of cancer-related pain and, as a topical treatment for actinic keratosis, which is commonly viewed as a pre-cancerous lesion. As these are licensed and common uses of DCF they are outside of the scope of this paper, except in the case where topical DCF is being investigated for other cancer indications.

## Current usage

### Dosage

DCF, which is available as a sodium or potassium salt, is used in tablet, gel/emulsion, injection and suppository forms. Dosages vary by format and indication. Typical doses for rheumatic disease and musculoskeletal disorders are in the range 75–150 mg in 2–3 divided doses, orally or rectally. Post-operative pain may be treated with diclofenac injections, either deep intramuscularly or intravenously, at a dose of 75–150 mg, with a maximum of 150 mg in 24 hours. The gel formulation utilises diclofenac sodium 3% in a sodium hyaluronate base and is applied twice daily for 60–90 days in the treatment of actinic keratosis [1].

### Toxicity

While it is a non-selective inhibitor of both isoforms of the cyclooxygenase enzyme (COX-1 and COX-2), DCF has a preferential binding to COX-2 [2], which may explain its intermediate risk profile for gastro-intestinal (GI) events in comparison with some other NSAIDs. Common side effects include abdominal pain, constipation, diarrhoea, dyspepsia, flatulence, heartburn, nausea and headache. Less common side effects include rash, itching, bloating, GI ulceration, oedema and dizziness. Rare but serious adverse events include GI bleeding, anaemia, liver failure, pancreatitis and pneumonia. As with all NSAIDs, long-term use DCF is also associated with a small increase in the risk of cardiovascular events, particularly myocardial infarction and stroke. A recent meta-analysis reported that the vascular risks of long-term DCF use were similar to those of selective COX-2 inhibitors, with a rate ratio of 1.41 for vascular events for DCF, compared to 1.37 for selective COX-2 inhibitors and 1.44 for ibuprofen [3].

DCF is contra-indicated in patients with previous history of hypersensitivity to aspirin or any other NSAID, suffering from congestive heart failure, ulcerative colitis or other inflammatory bowel condition, active GI ulcer or bleeding. It is also recommended that DCF be avoided in the final trimester of pregnancy and caution be exercised during lactation.

### Pharmacokinetics

Oral DCF is rapidly absorbed and almost completely distributed to plasma and tissues with little evidence of drug accumulation after repeated dosing within the normal therapeutic range [4–6]. Peak plasma concentration following a single 50 mg enteric coated diclofenac sodium tablet is 5.0 μM, attained in around 2 hours. The potassium salt of DCF is absorbed more rapidly, and a 50 mg tablet reaches a peak plasma concentration of 3.8 μM in 20–60 minutes. Terminal half-life is 1.8 hours after oral dosing. About 60% of the drug and its metabolites are eliminated in the urine and the balance through bile in the faeces. More than 90% of an oral dose is accounted for in elimination products within 72 hours, with only 1% of an oral dose excreted as unchanged parent compound in urine.

## Pre-clinical evidence in cancer - *In Vitro* and *In Vivo*

This paper focuses on the evidence of an anticancer effect of DCF treatment, including data that is specific to DCF and other data that is in line with DCF’s effects as an NSAID. In particular the emphasis is on DCF treatment post-diagnosis rather than on evidence of efficacy in a cancer chemo-prevention context (see [7] and [8] for reviews on NSAIDs in chemo-prevention).

### Fibrosarcoma

The first evidence for a possible anti-tumour effect of DCF was shown in experimental studies in implanted tumours (fibrosarcoma and hepatoma) in a rat model in 1983 [9]. Chemically induced rat tumours treated with a range of prostaglandin synthase inhibitors (indomethacin, diclofenac and aspirin) showed reduced growth rate and levels of vascularisation. Subsequent work indicated that DCF increased tumour blood flow *in vivo*, possibly via a role for prostaglandins in vascular permeability [10].

The *in vivo* effect of DCF on implanted fibrosarcoma tumours in mice was confirmed in 2002 by Hofer and colleagues [11, 12]. G:5:113 murine fibrosarcoma cells were implanted in male C3H/DiSn mice and tumours allowed to develop for five days before drug treatment with DCF, ibuprofen or flurbiprofen at an i.p. dose of 0.15 mg/mouse commenced. Two regimens were used, drug for five days (regimen A) and for fourteen days (regimen B) continuously and animals were monitored for a period of 15 weeks. The dose was selected by authors as it approximated the typical human NSAID dose of 1–2.5 mg/kg/day. Regimen B was superior to regimen A for all three drugs tested. Tumour growth inhibition in DCF treated animals was observed three weeks after application in regimen B (46% of control values, *P* = 0.019). *In vitro* analysis showed a decrease in cell numbers in response to DCF concentrations of 5 μM, 10 μM and 20 μM.

### Colorectal cancer

The anti-proliferative effects of a range of NSAIDs, including DCF, were assessed in three human colon cancer cell lines (HT-29, SW480, and DLD-1) *in vitro* in 1994 [13]. DCF was found to exert an anti-proliferative effect and had an IC50 of 55 μM, 37 μM and 170 μM respectively, making DCF one of the most potent of the panel of drugs tested. Later investigators studied the *in vivo* effect of topical application of DCF with hyaluronan on implanted colon-26 adenocarcinoma tumours in a BALB/c murine model [14]. Topical application at a dose of 6 mg/kg retarded and then stopped tumour growth compared to controls.

Additional evidence for an effect in colon cancer cell lines came from a study in 2003 by Falkowski and colleagues [15]. In addition to *in vitro* results which showed that DCF had a dose dependent effect on the C-26 murine colon adenocarcinoma cell lines, the authors also treated Balb/c x C57 BL/6 mice bearing syngeneic colon tumours with DCF at the dose of 250 mg/L in drinking water. Treatment commenced four days after tumour cell implantation and proceeded for 12 days. Tumour growth was reduced compared to untreated controls by day four of treatment and continued until treatment end. Prostaglandin E2 (PGE_2_) and thromboxane B2 (TBX_2_), both metabolites involved in the arachidonic acid cascade, were also significantly reduced by treatment end.

DCF is a component of the anti-angiogenic combinational drug combination TL-118, the other components being cimetidine, low dose cyclophosphamide and sulfasalazine. The efficacy of TL-118 was investigated in a mouse model of liver metastases from colorectal cancer and compared to treatment with rapamycin and the B20 anti-VEGF antibody [16]. CT-26-murine colorectal adenocarcinoma cells were injected into the spleen of male CB6F1 mice, leading to the formation of hepatic tumour nodules within 17 days of inoculation. Mice were treated with TL-118 intraperitoneally. The DCF dose is reported as 30 mg/kg, and is included in the TL-118 protocol on days one and four of each six day cycle of treatment. Only mice treated with TL-118 showed significant tumour growth delay, with both partial and complete remissions recorded. Overall survival in both partial and complete remission groups was significantly longer than untreated controls and animals treated rapamycin and B20 (*P* < 0.0005).

### Neuroblastoma

DCF was also tested for activity in neuroblastoma cell lines and xenograft models [17, 18]. Johnsen *et al* showed that COX-2 was over-expressed in 27 of 28 (96%) tissue samples from paediatric neuroblastoma patients, with no staining in surrounding tissues. *In vitro* DCF and the selective COX-2 inhibitor celecoxib inhibited cell growth in a panel of neuroblastoma cell lines. The IC50 ranged from 12.5 to 50 μM for celecoxib and 100 to 600 μM for DCF, with evidence of increased apoptosis in response to DCF. *In vivo* nude rats carrying SH-SY5Y neuroblastoma xenografts were treated with DCF in drinking water at a dose of 200 mg/L or 250 mg/L. Tumour growth was significantly inhibited after 2 days of DCF treatment (200 mg/L, *P* = 0.042; 250 mg/L, *P* = 0.024) compared with untreated controls. At the higher dose tumour growth was inhibited throughout the treatment period (11 days after the appearance of palpable tumours), and tumour weight at autopsy was lower than untreated controls for both doses, (median tumour weight 1.52 g, 0.22 g and 0.21 g for control, 200 mg/L and 250 mg/L groups respectively, *P* = 0.009).

In their most recent work, this same group of investigators have identified a high-risk, inflammatory subset of neuroblastomas associated with deletion of chromosome 11q [19]. In addition to analysis of patient samples, an *in vivo* model was used (nude mice inoculated with SK-N-AS cells harbouring an 11q-deletion) to test the effect of DCF on tumour growth. Mice were treated with DCF at a dose of 250 mg/L in drinking water. The difference in tumour volume between DCF-treated mice and controls was significant by day 8 (approximately 33% lower, *P* = 0.01) and day 9 (approximately 40% lower, *P* = 0.008).

The TL-118 drug combination was also tested against neuroblastoma [20]. An aggressive orthotopic model was generated by implanting human SK-N-BE (2) cells into NOD-SCID mice. TL-118, was tested alone and in combination with gemcitabine or 13-cis-retinoic acid. Initial testing with TL-118 showed a high level of toxicity and therefore a reduced dose was used. TL-118 alone reduced tumour growth rate and extended survival 1.5-fold (*P* < 0.0001). Treatment with gemcitabine alone also extended survival, but in combination with TL-118 the effect was significantly more pronounced, increasing survival 2.5-fold (*P* < 0.001), suggesting a synergistic effect.

### Ovarian cancer

Zerbini and colleagues assessed the combinatorial effect of combination treatment with NSAIDs in ovarian cancer cell lines and *in vivo* [21, 22]. The effect of a panel of NSAIDs was tested against four ovarian cancer cell lines (SKOV-3, CAOV-3, SW626 and 36M2) and assessed singly and, for the most potent, in combination. DCF (in the range 20–200 μM) and sulindac sulphide showed the strongest activity, inducing apoptosis and inhibiting cell growth in all four cell lines. The combinations of DCF plus sulindac sulphide, DCF plus naproxen, sulindac sulphide plus naproxen and sulindac plus ebselen were more effective than single drug treatments. *In vivo* SCID mice were injected with SKOV-3 cancer cells and were fed a control diet or diet supplemented with DCF (dose 100 ppm) or sulindac sulphide (dose 200 ppm). All mice developed tumours, but DCF or sulindac-fed mice bore tumours with volume 20% or 30% lower than controls (*P* < 0.05).

Later work by Valle and colleagues also investigated the use of NSAIDs, specifically DCF and indomethacin, in ovarian cancer cell lines and an *in vivo* model [23]. Serous ovarian adenocarcinoma cell lines HEY, OVCAR5 and UCI-101 were treated with varying concentrations of the two NSAIDs in the range 0–500 μM for 24 hours and assessed for cell viability. DCF treatment significantly (*P* < 0.05) reduced cell viability at concentrations of 50 μM in the HEY and OVCAR5 cells, and at 250 μM in the UCl-101 line. The HEY cell line was used in the *in vivo* experiments in athymic nude mice. For the DCF group treatment commenced 3 days after inoculation, DCF was administered intraperitoneally twice a week for four weeks at a dose of 18 mg/kg. DCF treatment reduced tumour growth compared to controls by 33% (*P* = 0.016), whereas treatment with indomethacin showed reduced tumour growth of 22% compared to their control group (*P* = 0.031).

### Other cancers

Results in an orthotopic syngeneic murine pancreatic cancer model showed that DCF treatment inhibited tumour growth compared to untreated controls [24]. Mice inoculated with PANC02 cells developed pancreatic tumours which readily metastasised to the peritoneal area around the incision site for tumour cell inoculation. Treatment with DCF was at a dose of 30 mg/kg (animal weight) given orally in drinking water and commenced three days after inoculation and continued for 11 days. Treated mice developed primary tumours with a mean weight 60% lower than untreated controls (*P* ≤ 0.01), while mean weight of metastatic tumours was also lower in DCF animals but the difference did not reach statistical significance. Analysis of tumour samples showed evidence of increased apoptosis and decreased angiogenesis compared to controls. However, *in vitro* experiments did not show evidence of apoptotic effect in PANC02 cells cultured with DCF at a concentration of 10 μM and 50 μM for four days.

DCF was also used in a murine glioma model [25]. The effect of increasing DCF concentrations between 100 μM–600 μM was assessed in cultured GL261 glioma cells. DCF below 200 μM impaired cell growth and concentrations above 300 μM caused cell death.

Lactate production by cells was significantly reduced at a concentration of 100 μM. *In vivo* female C57BL/6 mice were orthotopically inoculated with GL261 glioma cells and then treated with DCF (at a dose of 25 mg/kg). As with the *in vitro* analysis, DCF decreased lactate production compared to controls, though not to a statistically significant level. However, DCF-treated mice had a statistically significantly higher median overall survival than control mice (30.5 days versus 24 days, *P* = 0.0156). Concurrent treatment of DCF and R848 (the TLR7/8 agonist resiquimod) did not show improvement in survival compared to either treatment alone. Subsequent work has shown that the IC50 for DCF treatment against a panel of human glioblastoma cell lines (HTZ-349, U87MG, and A172) is in the range 50–200 μM, which are physiologically relevant [26].

The combination of DCF and sorafenib was the subject of an *in vitro* study using a panel of nine melanoma cell lines [27]. The combination had been selected after a functional screen to identify promising synergistic combinations of drugs with activity against melanoma cell lines displaying the major genetic drivers of the disease (BRAF, NRAS, CDKN2A etc). Combination treatment of sorafenib and DCF was effective against all cell lines, regardless of genotypic status. A different research group also investigated the *in vitro* activity of sorafenib and a number of COX inhibitors, including DCF, in the HepG2 hepatocellular carcinoma (HCC) cell line [28]. The results showed that DCF treatment at a concentration of 50 μg/mL significantly reduced proliferation (*P* < 0.01).

*In vivo* evidence for an effect of DCF in melanoma has also been published. Gottfried *et al* investigated the effect of DCF on the Myc transcription factor and glucose metabolism in leukaemia, prostate cancer and melanoma cell lines [29]. Additionally the group studied the effect of DCF in a syngeneic murine model, (C57/BL6 mice inoculated with B16 melanoma cells). Fourteen days post-inoculation DCF intraperitoneal treatment commenced at a dose of 15 mg/kg. A significant growth inhibitory effect was apparent within three days compared to controls (*P* < 0.05), and tumour weight and volume were significantly reduced (*P* < 0.001) at the end of the experiment (23 days after tumour inoculation).

Inoue and colleagues investigated the *in vivo* and *in vitro* effect of topical DCF application in prostate cancer [30]. Using two prostate cancer cell lines, one of which was transfected to over-express COX-2, the investigators treated each line with varying concentrations of DCF in the range 0 to 1000 μM for 72-hours. There was a dose dependent reduction in cell viability, with the COX-2 cells more sensitive to DCF. Cell viability in the COX-2 versus non-COX-2 cell lines was 74.0% and 95.7% (*P* = 0.0094), 51.6% and 73.8% at 50 μM (*P* < 0.0001) respectively. The IC50 was calculated as 42.2 μM and 91.6 μM, respectively. Further *in vitro* experiments showed that the cells over-expressing COX-2 were more resistant to radiotherapy than non-COX-2 over-expressing cells. The addition of DCF to cultures increased the effect of radiotherapy in the COX-2 cell line, significantly decreasing the survival fraction at a 2 Gy dose from 78.6% to 35.5% (*P* = 0.0225). This effect was confirmed in a xenograft model, with male BALB/c nu/nu mice inoculated with the COX-2 over-expressing cell line and treated when tumours reached 0.5 cm in diameter. In addition to untreated controls, three treatment groups were used, topical DCF alone, radiotherapy alone and topical DCF + radiotherapy (at a dose of 3 Gy). On day 36 following treatment, the mean tumour volume for the DCF group was 32% of the control group, 44% for radiotherapy group and 15% for the combination group (all *P* < 0.05).

## Human data

In contrast to the wide range of *in vitro* and *in vivo* results, there is a relative paucity of clinical data with respect to the use of DCF as an anticancer agent rather than as an analgesic. While there has been much clinical interest in the use of NSAIDs in cancer therapy, much of this has been focused on selective COX-2 inhibitors such as celecoxib. However, data is not completely lacking and is outlined below.

Forget and colleagues reported on a retrospective analysis of breast cancer patients treated with conservative surgery, with and without intraoperative NSAIDs (DCF or ketorolac) [31]. Patients treated pre-incisionally with ketorolac (20 mg -30 mg) or DCF (75 mg) showed improved DFS (HR = 0.57, 95% confidence interval CI: 0.37–0.89, *P* = 0.01) and an improved OS (HR = 0.35, CI: 0.17–0.70, *P* = 0.03), compared to patients not treated with NSAIDs. The proportion of NSAID-treated patients who received DCF was 29% (147/510), which was insufficient for an adequately powered analysis of DCF, therefore the data for DCF and ketorolac was pooled in the study. Subsequently a Phase III prospective randomised trial using ketorolac has been instituted (NCT01806259) to investigate the impact on distant relapse and overall survival of patients undergoing breast cancer surgery [32]. Accrual for this trial completed in August 2015 and primary end-point analysis (recurrence-free survival) is due in September 2017 (personal communication, Patrice Forget).

A similar retrospective study, by the same authors, looked at cohorts of patients for cancers of the breast, lung and kidney who had undergone surgical resection [33]. Of note results in non-small cell lung cancer (NSCLC) showed a statistically significant impact of pre-operative DCF on the risk of distant metastases (HR = 0.14 CI = 0.02–0.99, *P* = 0.05) and a tendency to improved mortality risk (HR = 0.61, CI: 0.35–1.06, *P* = 0.08) compared to no NSAID use.

As mentioned previously, TL-118 is a four-drug combination treatment that includes DCF. It is produced by Tiltan Pharma Ltd, Israel. A report was published outlining the use of standard of care chemotherapy (gemcitabine) and long-term use of TL-118 in a case of inoperable pancreatic adenocarcinoma, (not biopsy-confirmed) [34]. Initial treatment was associated with a sustained reduction in the CA 19 -9 tumour marker and a radiologically confirmed near-complete remission. TL-118 treatment was suspended due to toxicity, (weakness and vomiting), followed by a later suspension of gemcitabine treatment, during which time serum CA 19-9 increased. Gemcitabine was re-introduced but CA 19-9 continued to rise. Re-introduction of TL-118 caused a sharp reduction of CA 19-9 again. The patient was still under combined treatment of gemcitabine and TL-118 and showing progression-free response 16 months post-diagnosis. By way of comparison the authors quote figures of 6 and 9 months as averages for progression-free survival and overall survival for patients treated with first-line chemotherapy for pancreatic cancer.

Desmoid tumours, also known as aggressive fibromatoses, are rare non-metastasising tumours that arise from fibroblastic cells. While they do not metastasise they are locally invasive and are treated with surgical resection where possible, however, recurrence is a frequent event. Lackner and colleagues reported on two cases of unresectable disease in paediatric patients who were treated with tamoxifen (at a dose of 1 mg/kg, orally, BID) and DCF (at a dose of 2 mg/kg, rectally, twice a day) [35]. Both patients exhibited long term (four years and two years) disease control. No toxicity was reported. The same authors subsequently reported positively on four additional patients in an 11-year observational study [36]. An additional case report from Teshima *et al* outlined a case of aggressive recurrent disease treated with DCF at an oral dose of 50 mg BID for a period of two years [37]. Long term treatment was without apparent toxicity and caused considerable reduction in tumour size and symptoms. Furthermore the positive effects were sustained even after cessation of treatment.

Inflammatory myofibroblastic tumour (IMT) is another rare soft tissue tumour that can be locally invasive and which has features of both benign and malignant disease. Standard treatment is surgical resection, but recurrent or non-resectable disease is common. Tao and colleagues reported on a rare case of retroperitoneal IMT which was not amenable to complete resection and was therefore successfully treated with a combination of methotrexate, cisplatin and DCF (dose unspecified) following tumour debulking [38].

## Clinical trials

As of 21st September 2015 there are four clinical trials on-going. Note that these trials are specifically looking for an anticancer effect from DCF or drug combinations including DCF. Trials for non-cancer indications or trials in cancer in which DCF is used for analgesia are not included.

NCT01935531 – This is a single-arm open label trial of topical DCF gel (3% DCF in 2.5% hyaluronic acid) in patients with actinic keratosis with the aim of assessing the impact of DCF on lactate production and tumour metabolism. Biopsy samples will be compared before and after three months of treatment. The primary outcome will be the level of lactate before and after treatment. Secondary outcomes include lactate levels in healthy skin in subset of patients, measures of metabolic change (e.g. glycolytic proteins, glucose levels etc).

There are also a number of clinical trials involving DCF as a component of TL-118, mentioned previously. The oral treatment is designed to be taken on six days out of seven: sulfasalazine is included on each treatment day, DCF and cyclophosphamide on days one and four and cimetidine on days two, three, five and six [16].

- NCT00684970 is a multi-centre Phase IIB trial for metastatic castration resistant prostate cancer. The primary end point is progression free survival from 24 weeks after commencement of treatment up to 3 years. Secondary end points include overall survival, time to PSA progression, PSA response and pain response in evaluable patients.
- NCT01509911 is an international multi-centre trial in metastatic pancreatic cancer for patients starting gemcitabine treatment. The primary outcome is the disease control rate after 16 weeks of treatment.
- NCT01659502 is a single centre study in pancreatic cancer. The primary outcome is clinical benefit measurement (a composite score based on pain, performance status and weight) in a two-year time frame.

## Mechanism of action

There are multiple mechanisms of action posited to explain the diverse anticancer effects of DCF. Many of these are common to other NSAIDs, particularly for COX-2 inhibitors such as celecoxib and similar drugs. Of particular significance in this respect is the role of the prostaglandins, especially PGE_2_. PGE_2_ is formed from the breakdown of arachidonic acid to prostaglandin H_2_ by COX-1 and COX-2 followed by further processing by microsomal prostaglandin synthase 1 (mPGES-1). Elevated levels of mPGES-1 and PGE_2_ are found in a range of different cancer types and are associated with the chronic inflammation that is associated with a pro-tumour microenvironment [39, 40]. DCF, in common with other inhibitors of the COX enzymes also acts to reduce PGE_2_ synthesis and therefore many of the anticancer effects of DCF are associated, directly or indirectly, with reductions in PGE_2_ levels. However, there is considerable variation in COX-1/COX-2 selectivity between different NSAIDs [2, 41], and some evidence that DCF binds to COX-2 via a different mechanism to other commonly used drugs [42], therefore in the discussion that follows DCF-specific evidence is referenced where available but in some cases reference is made to generic COX-2/PGE_2_ mechanisms.

Relevant mechanisms of action include:

- Anti-angiogenic
- Immunomodulation
- Pro-apoptotic
- Platelet function
- Actions on Myc and glucose metabolism
- Treatment Sensitivity

### Angiogenesis

Inhibition of tumour neo-angiogenesis was one of the earliest anticancer mechanisms identified for DCF. Early work by Peterson and colleagues, in 1983, established that administration of DCF to animal models of cancer (fibrosarcoma and hepatoma) diminished the growth rate and degree of vascularisation of tumours, as did indomethacin or aspirin [9]. Earlier work had already shown that indomethacin and aspirin had growth inhibitory effects in cancer, although initially it was hypothesised that this was primarily due to reversal of the immunosuppressive effects of PGE_2_ [43]. A purely immune-related explanation for the growth inhibition of indomethacin and aspirin was discounted experimentally and a number of alternative mechanisms investigated [44].

Both topical and oral administration of DCF was found to retard implanted colon-26 tumour growth in BALB/c mice with a corresponding decrease in tumour angiogenesis which was associated with a reduction of PGE_2_ synthesis [14]. One mechanistic explanation is that PGE_2_ upregulates the production of VEGF via the prostanoid E receptors (EP1 – EP4) [45, 46]. Indeed genetic deletion of mPGES-1 in MMTV/NDL mice, which are genetically predisposed to the development of HER2/neu breast cancer, showed reduced incidence of mammary tumours, reduced levels of PGE_2_ and VEGF-A expression, and lower levels of angiogenesis (measured as microvessel density) [47]. Similarly, analysis of high-risk neuroblastoma subsets by Larsson *et al*, showed that high mPGES-1 expression correlated with poor patient survival and that treatment with DCF down-regulated PGE_2_ and that this correlated with reduced tumour growth volumes in an *in vivo* murine model [19].

The effect of DCF on VEGF expression has also been directly assessed in a number of tumour types. For example, after analysing COX-1, COX-2, VEGF-A and VEGF-C expression in a large panel of oesophageal carcinoma tumour samples (*n* = 123), von Rahden *et al* assessed the effect of three COX inhibitors, including DCF, in three oesophageal carcinoma cell lines (OSC-1, OSC-2 and OE-33) [48]. Treatment with DCF, at a concentration of 10 μM, significantly reduced expression of VEGF-A after 6 hours exposure in the OSC-1 and OSC-2 cell lines, and reduced expression of VEGF-C after 6 and 12 hours exposure respectively in the OSC-1 and OE-33 cell lines.

Similarly, Mayorek and colleagues compared VEGF levels in mice bearing orthotopic syngeneic pancreatic tumours treated with DCF and untreated controls [24]. Mice treated with DCF at a dose of 30 mg/kg/day, administered in drinking water starting 3 days after inoculation with PANC02 cells, developed tumours 60% lower in weight than in untreated controls. Tumours from DCF-treated animals also showed significantly lower levels of VEGF expression and lower levels of VEGF in peritoneal fluid, however plasma VEGF levels showed no difference. Ex-vivo analysis using rat aortic rings treated with DCF at a concentration of 10 μM showed that sprouting area was inhibited 2.5 fold compared to untreated controls.

In addition to VEGF, DCF may affect other angiogenic pathways. Kaur and Sanyal investigated the role of DCF in a chemically-induced murine colorectal cancer model [49]. In addition to down-regulation of VEGF, they investigated the role of two chemokines, monocyte chemoattractant protein (MCP-1) and macrophage inflammatory protein (MIP-1α), in angiogenesis. Treatment of animals with a known carcinogenic agent (1, 2-Dimethylhydrazine) with and without co-administration of DCF, at an oral dose of 8 mg/kg/day, showed that DCF reduced VEGF expression. DCF also decreased MCP-1 expression, another known marker of angiogenesis. However, DCF increased the expression of MIP-1α (aka CCL3) which is involved in both inflammatory and angiogenic processes.

The pro-angiogenic factors VEGF and basic fibroblast growth factor (bFGF) enhance the expression of the CXCR4 chemokine receptor on endothelial cells rendering them more responsive to CXCL12 (also known as stromal-derived factor 1α) signalling, associated with both increased angiogenesis and the metastatic cascade [50]. This process can be upregulated by PGE_2_, and it has been shown that reducing its expression using the COX-2 inhibitors piroxicam and NS398, it is possible to reduce CXCR4 expression and subsequent level of angiogenesis by 50–60% in an *in vivo* matrigel plug experiment [51].

Furthermore, Colleselli *et al* showed that COX-2 had an effect on endothelial progenitor cells (EPC), which are mobilised from the bone marrow and are involved in tumour angiogenesis [52]. Of the two co-enzymes COX-2 inhibition was associated with a greater reduction in EPC proliferation and an increase in the rate of apoptosis. DCF, at a concentration of 10 μM, produced a statistically significant decrease in EPC numbers, a result in line with that for celecoxib but not for the COX-1 inhibitor acetylsalicylic acid. In terms of apoptosis, DCF at a concentration of 50 μM and celecoxib at a concentration of 25 μM produced statistically significant increases in apoptosis compared to controls.

### Immunomodulation

In addition to having pro-angiogenic effects, it is known that tumour-associated PGE_2_ has negative effects on anti-tumour immunity [39, 53]. In addition to *in vitro* and *in vivo* evidence of immunosuppressive effects, there is also evidence from patient samples which correlate COX-2/PGE_2_ expression with immunosuppression in a number of different cancers [54–56]. The effects of prostaglandin inhibition was also investigated *in vitro* in lymphocyte subsets derived from breast cancer patients [57, 58]. Analysis had shown that irradiation was associated with immunosuppression via reduction in mitogen response in lymphocyte populations in response to increased prostaglandin synthesis. In vitro treatment with a range of prostaglandin inhibitors reversed this effect and could enhance mitogen responses, with DCF having the most potent effect.

While tumour-associated immunosuppression is a complex and multi-factorial process, a number of immune cell sub-populations are particularly implicated, including myeloid derived suppressor cells (MDSCs) and regulatory T cells (T-reg).

PGE_2_ has been shown to induce the differentiation of bone marrow stem cells into MDSCs in a number of animal models of cancer. For example Sinha *et al* showed that BALB/c mice carrying 4T1 mammary carcinomas had delayed tumour growth and reduced MDSC populations when the EP2 PGE_2_ receptor was knocked out, compared with wild-type mice [59, 60]. Treatment of wild-type mice carrying 4T1 tumours with a COX-2 inhibitor (SC58236) also reduced tumour growth rates and reduced the accumulation of MDSC cells. Similarly, Fujita and colleagues showed that in a mouse model of glioma COX-2 blockade using aspirin or celecoxib inhibited PGE_2_ production and delayed tumour progression [61]. This was associated with reduced accumulation of granulocytic MDSCs and an increased presence of cytotoxic T lymphocytes (CTLs). Of note it was also reported that treatment with aspirin was only effective if administered prior to tumour development, whereas the selective COX-2 inhibitor celecoxib was effective when administered starting 21-days post-tumour implantation. The celecoxib dose used was 30 mg/kg/d, which the authors reported as equivalent to a human dose of 400 mg/day, which is a clinically relevant dose. Finally, Veltman *et al* used a murine model of mesothelioma and showed that treatment with dietary celecoxib reduced the local and systemic expansion of MDSC sub-populations, and that this correlated with a reduction in immune suppression [62].

In addition to animal models of cancer-related MDSCs there is some data from ex-vivo patient samples presented by Mao *et al* [63]. Different populations of mononuclear cells were isolated from melanoma patients and tested for immunosuppressive activity. CD14^+^HLADR^low/-^ cells significantly inhibited the cytolytic activity and IFNγ production of autologous non-activated natural killer (NK) cells, with the effect due to release of TGFβ. PGE_2_ enhanced the production of TGFβ by monocytic cells. Using a mouse model the authors showed that abrogation of tumour COX-2 expression reversed immunosuppression and increased the lytic activity of NK cells.

While there is no direct evidence for a DCF-specific action on MDSCs, there is little doubt that it is a potent inhibitor of PGE_2_ [64, 65]. Similarly, as we have seen above there is evidence that a range of selective and non-selective COX-2/PGE_2_ inhibitors can reduce MDSC populations, and that therefore we would expect similar activity from DCF.

Certain regulatory T cell populations are also known to be associated with tumour-associated immunosuppression, particularly CD4^+^CD25^+^FOXP3^+^ cells [66–68]. Of particular interest is the role of COX-2/PGE_2_ in the increase in T-reg cell numbers and immunosuppressive phenotype, which has been established in a number of tumour models [53, 56, 69, 70]. Reduction of tumour-induced PGE_2_ using both selective and non-selective COX inhibitors has been shown to reduce T-reg populations and activity [71–74]. In terms of DCF-specific evidence, Chirasani and colleagues showed both *in vitro* and *in vivo* that DCF was able to reduce the intra-tumoural accumulation and activation of T-regs in a murine glioblastoma model [25]. DCF, at a concentration of 1.5 μM, was also used *in vitro* to reduce the suppressive activity of T-reg of head and neck squamous cell carcinoma cell lines [70].

PGE_2_ is also implicated in tumour-associated immunosuppression via inhibition of antigen presenting cells (APC)/dendritic cell (DC) induction and maturation. In addition to evidence from primary tumour cultures [75], there is also evidence that some stromal cell populations also secrete PGE_2_ and are involved in inhibition of DC maturation [76]. Eruslanov *et al* showed in an *in vitro* study that PGE_2_ skewed the differentiation of Th1 APCs towards MDSCs or tolerogenic M2-polarised macrophages [77]. It was further shown that co-culture with the COX-2 inhibitor LM-1685 partially restored expression of CD11c, a DC marker.

However, there is also some evidence that PGE_2_ is required for activated DC migration to lymphoid tissues [78, 79]. Yen and colleagues used an *in vivo* model to show that DCs matured within inflammatory sites require both CCR7 and PGE_2_-induced MMP-9 for their directional migration to draining lymph nodes [79]. There are, therefore, both immunostimulatory and immunosuppressive roles for PGE_2_ in the full life-cycle of DCs [80].

Analysis by Trabanelli *et al* suggests that the disappointing clinical responses of DC vaccines in oncology are due to the induction of tolerogenic responses mediated by PGE_2_, and that the positive effects of PGE_2_ on DCs are mitigated by the upregulation of the immunosuppressive enzyme indoleamine 2, 3-dioxygenase-1 (IDO1) [81]. The clinical implications of these diverse results are discussed in the Our Take section of this paper.

### Apoptosis

In addition to modulation of angiogenesis and immune suppression, there is some evidence for a pro-apoptotic mechanism of action for DCF in cancer. One of the earlier reports outlining an anticancer effect of topical DCF with hyaluronan showed that incubation of colon-26 cells with DCF at concentrations between 30 – 300 μM induced a significant increase in apoptosis [14]. Some indication that this effect was independent of COX-2/PGE_2_ inhibition was provided by Kusuhara and colleagues who showed that apoptosis induction by DCF in cultured rat gastric mucosa cells was associated with caspase-dependent DNA fragmentation [82, 83]. This finding was in line with similar contemporary reports using other NSAIDs, including sulindac [84], aspirin [85] and indomethacin [86]. In contrast, Ashton showed no such increase in apoptosis in guinea-pig gastric mucosal cells exposed to 250 μM for 24 hours [87].

Gardner and colleagues further elucidated the mechanism of action with respect to human colorectal cancer in a number of NSAIDs, including DCF [88]. Using the SW480 human colorectal cancer cell line, which does not express COX-2, they investigated the effects of indomethacin, sulindac sulphide, sulindac sulphone, rofecoxib and DCF on proliferation, apoptosis, β-catenin and cyclin-D1 *in vitro*. DCF, at a concentration of 200 μM, induced a statistically significant anti-proliferative effect but this was associated with a decrease in apoptosis. DCF was also associated with a decrease in β-catenin protein levels and cyclin D. Overall, DCF was shown to have moderate anti-proliferative and weak pro-apoptotic activity in this cell line. Additional *in vitro* confirmation of an inhibitory effect on β-catenin came from the work of Lu *et al* who confirmed that a panel of NSAIDs, including DCF, repressed β-catenin via high-level expression of peroxisome proliferator-activated receptor-γ (PPAR-γ) [89].

Inoue *et al* investigated the apoptotic activity of DCF in the HL-60 human promyelocytic leukaemia cell line [90]. DCF, at concentrations above 100 μM, induced DNA fragmentation and apoptosis, trigged the caspase cascade and release of cytochrome c. This was associated with an increase in intracellular reactive oxygen species (ROS), with a downstream inhibition in Akt phosphorylation via a PI3 kinase (PI3K) pathway. Johnsen *et al* also reported a pro-apoptotic effect of DCF in neuroblastoma, with *in vitro* analysis revealing evidence of DNA fragmentation and a caspase-dependent pathway [17]. Inhibition of Akt/PI3K signalling was also apparent in colorectal cancer in work by Rana *et al* [91]. Similarly, Albano *et al* reported that apoptosis in the human melanoma cell lines A2058 and SAN was associated with an increase in intracellular ROS and increase of caspase-9 and -3, reduction of Bcl-2/Bax ratio and mitochondrial release of cytochrome c [92].

Singh *et al* also reported on apoptosis in the leukemic cell lines HL-60 and THP-1, and in 43 samples from acute myeloid leukaemia patients [93]. Induction of apoptosis was via the activation of several AP-1 family transcription factors, (such as c-Jun, JunB and Fra-2), and subsequent induction of GADD45α which in turn activates JNK to trigger apoptosis.

Braun *et al* investigated the pro-apoptotic effects of DCF, acetylsalicylic acid (ASA) and sodium salicylate (NaS) on cutaneous T-cell lymphoma cell lines (CTCL) [94]. CTCL is a heterogeneous group of non-Hodgkin lymphomas that includes mycosis fungoides and Sézary syndrome, and CTCL cells are known to be resistant to apoptosis triggered via death receptors [95]. *In vivo* treatment with NSAIDs, including DCF at a concentration 200 μM, restored sensitivity to tumour necrosis factor-related apoptosis-inducing ligand (TRAIL)-induced apoptosis. In addition to *in vitro* analysis based on established CTCL cell lines, *ex vivo* analysis of T-cells from four Sézary syndrome patients showed enhanced apoptotic response in three of them compared to healthy controls. In addition to the work of Braun *et al*, other workers have also investigated the use of COX-2 inhibition in CTCL, for example *in vivo* work using celecoxib in a mouse model of mycosis fungoides [96].

Another COX-2/PGE_2_-independent pro-apoptotic pathway is via increased expression of non-steroidal anti-inflammatory drug-activated gene 1 (NAG-1), also known as macrophage inhibitory cytokine-1. NAG-1 is a member of the transforming growth factor-beta (TGF-β) superfamily with evidence of both pro- and anti-cancer activity, possibly related to stage of disease [97]. Over-expression of NAG-1 has been related to induction of apoptosis in a range of cancer types, possibly as a down-stream target of p53 signalling [97–100]. Kim *et al* tested the relative effect of different NSAIDs on the induction of NAG-1 in an oral squamous cell carcinoma line (SCC 1483) [101]. DCF was the most potent of the panel of NSAIDs used, increasing NAG-1 expression five-fold at a concentration of 100 μM. NAG-1 expression increased prior to the induction of apoptosis, with which it was highly correlated.

In a PANCO2 pancreatic cancer model, Mayorek and colleagues showed that DCF treatment at a dose of 30 mg/kg caused a 60% reduction in tumour weight compared to untreated controls, and that the reduction in tumour weight was caused by an increased rate of apoptosis [24]. The effect was not evident *in vitro* and further analysis showed both a reduced rate of angiogenesis and an increased level of arginase activity in tumour stroma and peritoneal macrophages. The increased level of arginase activity was associated with a reduction in nitric oxide (NO) and arginine depletion in the peritoneal cavity and serum, although it was not shown how this could enhance the apoptotic effect of DCF treatment.

Apoptosis due to NSAID treatment, including DCF, was also shown in a panel of ovarian cancer cell lines by Zerbini and colleagues [22]. The induction of apoptosis was mediated by the pro-apoptotic cytokine melanoma differentiation associated gene-7/Interleukin-24 (mda-7/IL-24), which mediates the induction of GADD45α expression and activation of the JNK pathway.

### Platelet function

There is increasing interest in the role of platelets in cancer, with emerging evidence of a role in tumour progression and metastasis. A number of mechanisms are known to be active in the pro-cancer role of platelets including the release of pro-angiogenic factors, ‘cloaking’ of tumour cells from NK cells and a role in preparing metastatic niches [102]. Cancer-associated thrombocytosis is a common clinical occurrence and is associated with poorer outcomes in a number of cancers [103–105]. Of note there is also some evidence that the putative anti-cancer effects of aspirin may be related to its anti-platelet effects via irreversible inhibition of COX-1 [106, 107]. Similarly there is some evidence that other anti-thrombotic therapies, for example low molecular weight heparins may also have anti-cancer or anti-metastatic activity [108, 109].

As a non-selective COX inhibitor, albeit with a preference for COX-2, DCF also has clinically relevant actions on platelet function via COX-1 inhibition. Van Hecken *et al* studied the effects of a panel of NSAIDS in healthy volunteers and found that at steady state dosing of DCF at 50 mg three times a day for six days *ex vivo* COX-1 level (expressed as thromboxane B2 generation in clotting whole blood) was reduced by 53% compared to base-line, and platelet aggregation was also significantly reduced (*P* < 0.001) [64]. This effect on platelet function has also been confirmed in a clinical setting, for example Bajaj *et al* reported a 64% reduction in platelet aggregation in patients treated with a single 75 mg dose of DCF administered pre-operatively [110].

We may speculate, therefore, that DCF may also exert anti-angiogenic, immunomodulatory and other anti-cancer effects via inhibition of platelet function in addition to COX-2/PGE_2_ mediated actions.

### Myc and glucose metabolism

There is also some evidence that DCF has an impact on tumour metabolism that is independent of its action as a COX-inhibitor. Gottfried and colleagues showed that DCF down-regulated Myc gene expression and glucose metabolism in a number of leukaemia, prostate cancer and melanoma cell lines *in vitro* and in an *in vivo* melanoma model [29]. Of note neither aspirin nor the COX-2 inhibitor NS-398 had an effect on Myc expression or glucose metabolism. Furthermore, DCF inhibited lactate efflux, causing an increase in cellular lactate levels which was independent of the effect on Myc gene expression. The increase in cellular lactate, leading to a decreasing proliferation rate, was also matched by a decrease in extra-cellular lactate. Similarly, COX-independent effects on lactate were reported in glioblastoma, both *in vivo* [25] and in human glioblastoma cell lines [26].

The effects on glucose metabolism may be related to impacts on glycolytic pathways via STAT3 inhibition [26], although there is some evidence that the effect may also be mediated via hypoxia-related down-regulation of glucose-transporter 1 (GLUT1) [111].

Further evidence for an effect on Myc expression was also provided by Sareddy and colleagues, who showed that DCF and celecoxib caused a reduced expression of Wnt/β-catenin/Tcf signalling in a two glioblastoma cell lines (U87 and U251) [112]. In vitro treatment showed that both drugs, (DCF concentration in the range 50–200 μM, celecoxib in the range 20–80 μM) significantly reduced expression of down-stream targets of β-catenin signalling, including c-Myc and cyclin D1. These effects were associated with reduced glioblastoma cell proliferation, colony formation and invasion.

### Treatment sensitivity

There is some evidence that COX-2 expression may correlate with sensitivity to chemotherapy or radiotherapy in different cancer types. For example, analysis of 104 cases of primary invasive breast cancer indicated that increased expression of COX-2 correlated, (*P* < 0.0001), with increased expression of multi-drug resistance gene (MDR1) and P-glycoprotein (P-gp), both mechanistically implicated in resistance to chemotherapy [113]. Furthermore expression of MDR1/P-gp had prognostic significance in terms of both PFS and OS in this patient population, (both *P* < 0.0001). In an analysis of advanced ovarian cancer cases, it was also found that COX-2 correlated to treatment resistance, (*P* = 0.0072), although this varied by chemotherapy drug, with resistance to platinum-based drugs more susceptible to COX-2 expression than treatment with paclitaxel.

Based on these and other results there has been an interest in the use of COX-2 inhibitors to potentiate sensitivity to chemotherapy [114, 115]. There has been limited clinical investigation of this strategy to date. A Phase II trial in heavily pre-treated ovarian cancer patients with recurrent disease, (*n* = 45, of whom 23 were platinum-resistant), showed that the combination of carboplatin and low-dose celecoxib (400 mg/day) had a response rate of 28.9%, including three complete regressions. Median PFS was 5 months overall, but among responders the PFS was 8 months and OS 17 months (statistical significance not shown) [116].

In chronic myeloid leukaemia (CML), the standard treatment for the chronic phase of the disease is with the targeted agent imatinib mesylate. Transport of the drug into CML cells is via the human organic cation transporter-1 (OCT-1) and low OCT-1 functional activity is associated with treatment resistance and poor patient outcomes [117]. In an investigation of drug-drug interactions between imatinib and a panel of common NSAIDs, DCF and ibuprofen were shown to have significant interactions [118]. Specifically, DCF at a clinically relevant concentration of 10 μM was shown to increase OCT-1 activity and to statistically significantly reduce the IC50 of imatinib in two CML cell lines (K562 and KU812). In contrast ibuprofen, at a clinically relevant concentration of 130 μM decreased OCT-1 activity and increased the IC50 value of imatinib. The use of DCF was also tested *ex vivo* using mononuclear cells (MNC) from newly diagnosed CML patients. DCF treatment in MNC samples with low baseline OCT-1 activity increased OCT-1 activity and reduced the IC50 of imatinib, clearly suggesting that DCF may be useful to sensitise patients at highest risk of treatment failure due to drug resistance.

Another mechanism of chemoresistance involves the repopulation of tumour masses by the accelerated proliferative response of cancer stem cells after chemotherapy, as has been shown by Kurtova *et al* in bladder cancer [119]. In vitro work showed that this process was driven by PGE_2_ and that it could be reversed by PGE_2_ inhibition. *In vivo* xenograft models of bladder carcinoma (T24 and a patient derived chemoresistant line), showed that with combined treatment of gemcitabine and celecoxib resistance to treatment did not occur.

Wasserman and colleagues investigated the long-term outcomes for women treated with adjuvant radiotherapy in the treatment of primary breast cancer [57]. They showed that local radiotherapy for breast cancer (45 Gy) caused a severe lymphopenia with reductions of both T- and non-T-lymphocyte counts and reactivity and that recovery of T-cells was still impacted 10–11 years after treatment. Furthermore mortality was greater in women with impaired T-cell function up to eight years after treatment. However, *in vitro* treatment with DCF showed improved reactivity of lymphocytes from radiation-treated women three months after the completion of their treatment.

Crokart *et al* investigated the effect of NSAID administration on oxygen pressure in two syngeneic murine cancer models (TLT liver tumours and FSaII fibrosarcomas) [120]. DCF, at a dose of 20 mg/kg by weight, showed an increase in tumour oxygen pressure around 30 minutes after administration, results which were similar to those for piroxicam and indomethacin. Further analysis using the COX-2 inhibitor NS-398 showed that this increase in oxygen pressure was associated with tumour regrowth delay in mice treated with radiotherapy.

## Our take

**Figure 1.**
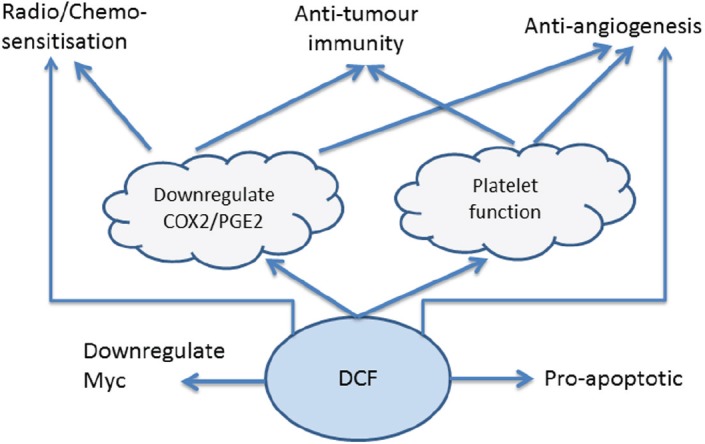
DCF mechanisms of action.

The pre-clinical and clinical data, summarised in Table 1, indicate that DCF has a number of distinct anti-cancer effects, summarised in Figure 1, which warrant further investigation in a clinical setting. These effects are mediated by both COX-dependent and independent mechanisms of action, suggesting that at least some of the effect is specific to DCF rather than being associated generically with other NSAIDs such as celecoxib, indomethacin and aspirin. DCF benefits from potent down-regulation of both COX-1 and COX-2, addressing both platelet- and PGE_2_-mediated pro-cancer pathways and also displays a range of COX-independent pathways. These multiple mechanisms of action make DCF one of the more interesting NSAIDs in the context of cancer treatment.

The combination of anti-angiogenic activity with positive effects on immunity is especially interesting in the context of surgical intervention in cancer. There is increasing evidence that the ‘wound healing’ response initiated by surgical intervention against tumours is implicated in distant metastatic relapse. Evidence for this effect comes both from retrospective analyses of patient outcomes and from *in vivo* models [121–123]. There are multiple mechanisms posited to be at work, many of them focused on the post-surgical inflammatory cascade leading to an up-regulation of angiogenic signalling and a sustained immune suppression [124–126]. In response there has been a new focus on those peri-operative interventions which may have an impact on the post-surgical relapse rate by selective targeting of aspects of this wound healing response, particularly with respect to the choice of anaesthesia [127–130]. A number of drugs have been identified which may have a positive effect when used in the peri-operative or post-operative setting, including ketorolac [32, 131, 132], cimetidine [133, 134], and DCF [31, 33]. Therefore, further investigation of the peri-operative use of DCF is warranted in a number of cancers in which post-surgical distant metastases are a frequent occurrence, including osteosarcoma, oesophageal carcinoma, NSCLC, ovarian and breast cancer.

The complex role that PGE_2_ plays in the complete life-cycle of DCs may mean that long-term use of DCF and COX-2 inhibitors may have some negative effects on anti-tumour immunity, although the picture remains unclear as to whether the effects are overall positive or negative. This suggests that caution may need to be exercised in the treatment schedule such that the negative effects are minimised and the positive maximised. Certainly the short-term use in peri-operative interventions may be positive precisely because of the timing of the treatment. Alternatively it is suggested by Trabanelli *et al* that blockade of IDO1 may also be a viable strategy to ameliorate the negative effects on DCs [81].

While there is some evidence that DCF has pro-apoptotic activity, much of this evidence is *in vitro* and uses relatively high drug concentrations. The current level of evidence does not support the use of DCF as an inducer of apoptosis and it is likely that the anticancer effects are primarily due to the other mechanisms of action.

We note also the strong pre-clinical evidence that DCF has an effect in neuroblastoma, a disease with a dismal prognosis for patients with refractory or metastatic disease. While there are a number of new targeted agents being investigated for this high-need population of patients, there are few clinical trials that have progressed to Phase III. The addition of DCF to either existing standard of care or with new targeted agents has the potential for clinical benefit and therefore warrants further investigation.

Additionally, given the potent effects that DCF has on PGE_2_ expression, there is merit in investigating the addition of DCF to existing standard of care therapy in those cancers in which PGE_2_ upregulation is associated, including breast, head and neck and colorectal cancers. For example, it is known that PGE_2_ promotes colorectal cancer growth via an upregulation of β-catenin signalling [135, 136], and that DCF can inhibit this *in vivo* [137].

Finally, while there have been a number of interesting and positive case reports of DCF activity against both desmoid and inflammatory myofibroblastic tumours, there have been no randomised clinical trials to confirm these results. Given the apparent low toxicity of DCF and the positive results that have been reported, investigation of DCF is clearly required, particularly as many of the agents currently being trialled, (examples include sorafenib, imatinib and crizotinib), have greater toxicity and costs associated with them.

### Next steps

The evidence is strongest for clinical trials of DCF, in combination with other agents, in the following cancer types:

- Desmoid tumours (metronomic treatment)
- Inflammatory myofibroblastic tumours
- High-risk refractory or metastatic neuroblastoma

The peri-operative use of DCF is also of interest in the following cancers:

- Osteosarcoma
- Head and neck cancers
- Oesophageal cancer
- Breast cancer
- Ovarian cancer
- Non-small Cell Lung Cancer

## Conclusion

Drawing on *in vitro*, *in vivo* and human data we have summarised the evidence for an anti-cancer effect of DCF treatment. The established pharmacokinetics and known toxicity profile make this generic drug a strong candidate for repurposing as an oncological treatment, both in combination with existing standard of care treatments or in a cocktail with other repurposed drugs. A number of possible multi-drug combinations are outlined in the supplementary materials for specific cancer indications.

## Author contributions

Primary author: Pan Pantziarka. Contributing authors: Vidula Sukhatme, Gauthier Bouche, Lydie Meheus, Vikas P. Sukhatme. All authors read and approved the final manuscript.

## Competing interests

The authors declare that they have no competing interests. All the authors are associated with not for profit organisations that aim to repurpose drugs for oncology treatments.

## Figures and Tables

**Table 1.** Summary of evidence by cancer type.

Cancer Type	In vitro	In vivo	Case Report/Trial
**Colorectal**	[13]	[14–16]	
**Neuroblastoma**	[17, 18]	[17, 18, 20]	
**Pancreatic**		[24]	NCT01509911 NCT01659502
**Ovarian**		[22, 23]	
**Glioma**	[112]	[25]	
**Melanoma**	[92]	[27, 29]	
**Prostate**		[30]	NCT00684970
**Breast**			[31]
**Desmoid Tumours**			[35–37]
**Inflammatory myofibroblastic tumour**			[38]

**Table 1.** Proposed drug combinations with DCF and standard of care in different cancers.

Disease	Targets	Drug Combination
**Colorectal cancer (resectable disease)**	Anti-angiogenic, immunomodulation, AMPK/mTOR	Pre-operative DCF Post-operative cimetidine [52] Aspirin (long-term) [53] Metfomin
**Bone/Soft-tissue sarcoma**	Reduction of post-surgical immune suppression, Hedgehog pathway, microtubule disruption, AMPK/mTOR	Pre-operative DCF Mebendazole [54] Itraconazole [55] Metformin Metronomic chemotherapy [56]
**Melanoma**	Invoke initial T-cell response, reverse resistance to immune checkpoint inhibitors	DCF Cimetidine Mebendazole [54, 57] Nivolumab Ipilimumab
**NSCLC**	Improve chemo-radiotherapy response, anti-angiogenic	DCF Nitroglycerin Plerixafor
**GBM**	PGE_2_ inhibition, Hedgehog pathway, microtubule disruption, autophagy inhibition	DCF Cimetidine Mebendazole [54] Plerixafor (during radiotherapy) Itraconazole
**Desmoid Tumours**	PGE_2_ inhibition, anti-angiogenic	DCF Tamoxifen [58]

Note that references to clinical trials or published papers are indicative of trials or case reports where the drug (or analogue) has been used for the specific indication.

## Appendix 1. Supplementary Material

### Introduction

The following drugs warrant further investigation in combination with diclofenac (DCF) and existing standard of care cancer treatments in a range of cancers. These combinations, listed in Table 1, have been selected on the basis of existing pre-clinical and clinical experience in each of the indications. In some cases these combinations replicate existing protocols currently being tested in clinical trials, but substitute known and repurposed drugs for the newer and/or more toxic agents currently being investigated. All of these proposed combinations are expected to display relatively low toxicity and use low cost and generally available agents. *The following drugs are not listed in order of priority*.

### Pharmacological interventions

The following proposed drug combination are primarily based on postulated synergies between DCF and other agents, including chemotherapeutics and a number of repurposed non-cancer drugs.

- Combination anti-PD1/anti-CTLA4 therapies – A major clinical focus in cancer immunotherapy is the use of immune checkpoint inhibitors, particularly anti-cytotoxic T-lymphocyte antigen 4 (anti-CTLA-4) and anti-programmed death-1 (anti-PD-1), to reverse immune suppression. Initial positive results using ipilimumab and nivolumab in melanoma have provoked interest in other cancer types and in the use of combination treatments [1–3]. However, despite some startling successes, some patients do not show a response, or acquire resistance to these therapies. There is some evidence that the degree of benefit may be associated with a pre-existing anti-tumour T-cell response [4, 5]. Spranger *et al* showed that in melanoma tumour-associated β-catenin signalling was associated with a lack of T-cell response and subsequent treatment failure in a murine model [6]. DCF has been shown to inhibit β-catenin signalling [7–9]. Additionally Zelenay *et al* showed that both aspirin and celecoxib synergised with anti-PD-1 blockade in murine models of melanoma and colorectal cancer [10]. Therefore the use of DCF, possibly in combination with other agents, in order to prime the T-cell response and improve the clinical benefit of checkpoint inhibitors is clearly warranted.
- Cimetidine – The H2 receptor antagonist cimetidine remains a widely used antacid for both short-term and long-term administration. There is a range of evidence, both pre-clinical and clinical, for a number of anticancer effects, particularly with respect to colorectal cancer [11, 12]. The anticancer mechanisms of action may be related to effects on cell adhesion, angiogenesis and a number of different effects on immunity. In respect to colorectal cancer the clinical evidence shows a positive effect on survival when used perioperatively for early stage colorectal cancer [11]. In one trial long-term use of cimetidine was associated with a significantly improved long-term survival in patients with colorectal cancer with high levels of sialyl Lewis-X and sialyl Lewis-A epitope expression on tumour cells [13]. The use of DCF pre-operatively and long-term cimetidine post-operatively warrants clinical investigation.
- Itraconazole – Itraconazole, a generic broad-spectrum anti-fungal drug, has been shown to have potent anticancer effects in a range of cancers [14]. The main mechanisms of action which have been posited include Hedgehog pathway inhibition, anti-angiogenic activity and reversal of multi-drug resistance. There is some evidence that itraconazole also targets the cancer stem cell (CSC) fraction in multiple myeloma [15]. Recent evidence suggests that elevated expression of COX-2 may be related to resistance via CSC populations in colorectal cancer [16]. DCF has also shown interesting activity in blocking CSC repopulation of tumour masses following debulking in a bladder cancer model [17]. Combination therapies which target the stem cell populations with agents such as DCF and itraconazole in addition to therapies targeting the non-CSC populations warrant additional pre-clinical and clinical study.
- Metronomic chemotherapy – Metronomic chemotherapy is an alternative to maximum tolerated dose (MTD) chemotherapy and is characterised by frequent, low-dose administration of many standard chemotherapy drugs. Where MTD chemotherapy aims to maximise tumour kill rates, metronomic chemotherapy appears to deliver its therapeutic effects via anti-angiogenic and immunomodulatory activities – with an attendant reduction in adverse effects and an improved quality of life [18–20]. To date a broad range of chemotherapy agents have been used metronomically, mostly via oral formulations, including cyclophosphamide, capecitabine, methotrexate, etoposide, vinorelbine and temozolomide [21]. There is much interest in the addition of further anti-angiogenic agents in combination with metronomics, with a number of trials using celecoxib in this respect [22–24]. As there is evidence that DCF has both anti-angiogenic and immunomodulatory effects the combination with metronomic chemotherapy is worthy of further clinical investigation, particularly in those cancers with high unmet needs, such as soft tissue sarcoma, metastatic breast cancer and ovarian cancer.

### Non-pharmacological interventions

DCF co-treatment may be a valid addition to a number of non-pharmaceutical cancer treatment modalities.

- Photodynamic Therapy (PDT) – PDT is a locally ablative cancer treatment that involves the administration of a photosensitive agent which preferentially accumulates in tumour tissue, followed by the application of a light source of specific frequency to the tumour such that the photosensitive agent reacts and causes tumour cell death via both apoptosis and necrosis. A number of studies have suggested that PGE_2_ is associated with increased resistance and cell survival in PDT [25, 26]. Therefore inhibition of COX-2/PGE_2_ has been investigated as a mechanism to improve response to treatment, primarily focusing on COX-2 inhibitors such as celecoxib and NS-398 [27–29]. While PDT is a local treatment, there is interest in the systemic immune effects which treatment is known to elicit and in particular with the development of protocols which increase anti-tumour immunity [30, 31]. The combination of PDT with short-term DCF treatment may serve to both reduce treatment resistance by inhibition of PGE_2_ and improve the systemic immune response.
- Perioperative – There is evidence that the perioperative period following primary tumour resection may be associated with an increased risk of distant metastases in a range of tumour types [32–34]. A number of mechanisms of action have been posited, with evidence in particular that the post-surgical immunosuppression associated with the inflammatory wound-healing response is involved [35, 36]. There is evidence from retrospective studies that the recurrence rate may be modulated by the use of NSAIDs, specifically ketorolac and DCF [37–40]. Two Phase 3 prospective clinical trials of pre-incisional ketorolac are currently underway in breast cancer (NCT01806259 [41] and NCT02141139). Additional trials are warranted in other cancers, including osteosarcoma, non-small cell lung cancer and oesophageal cancer. In addition to ketorolac there is sufficient data to suggest that other agents may be of some value in the perioperative setting, including DCF, cimetidine, Polysaccharide-K (PSK) and other agents, alone or in combinations.
- Radiotherapy – Radiotherapy remains a key treatment modality in many forms of cancer, with much interest in the development of new agents to improve therapeutic response [42]. COX-2/PGE_2_ inhibition has been explored as a potential radiosensitisation strategy in a number of tumour models, with positive results [43–46]. There have been a number of small clinical trials exploring this strategy in a range of cancers, including NSCLC [47], nasopharyngeal carcinoma [48], head and neck cancer [49] and other malignancies. Given the relative COX-2 selectivity of DCF, clinical exploration may be warranted, particularly in the case of intraoperative radiotherapy regimens where DCF may provide positive effects both in radiosensitisation and in reversing the post-surgical inflammatory response. In the case of glioblastoma multiforme (GBM), there is evidence that post-irradiation recurrence is associated with hypoxia-induced regrowth of damaged vasculature, a process mediated by SDF-1/CXCR4 interaction and the recruitment of bone-marrow derived cells [50]. Inhibition of SDF-1/CXCR4 interactions using plerixafor has been shown to inhibit the vasculogenic cascade. A combination of plerixafor with DCF, noting that PGE_2_ inhibition can also down-regulate CXCR4-related angiogenesis [51], would therefore be of some interest in the case of GBM radiotherapy.

## References

[1] Joint_Formulary_Committee (2015). British national formulary.

[2] Cryer B, Feldman M (1998). Cyclooxygenase-1 and cyclooxygenase-2 selectivity of widely used nonsteroidal anti-inflammatory drugs. Am J Med.

[3] Baigent C, Bhala N, Emberson J (2013). Vascular and upper gastrointestinal effects of non-steroidal anti-inflammatory drugs: meta-analyses of individual participant data from randomised trials. The Lancet.

[4] Davies NM, Anderson KE (1997). Clinical pharmacokinetics of diclofenac Therapeutic insights and pitfalls. Clin Pharmacokinet.

[5] Gan TJ (2010). Diclofenac: an update on its mechanism of action and safety profile. Curr Med Res Opin.

[6] Su SF, Chou CH, Kung CF (2003). In vitro and in vivo comparison of two diclofenac sodium sustained release oral formulations. Int J Pharm.

[7] Fischer SM, Hawk ET, Lubet RA (2011). Coxibs and other nonsteroidal anti-inflammatory drugs in animal models of cancer chemoprevention. Cancer Prev Res.

[8] Gurpinar E, Grizzle WE, Piazza GA (2013). COX-independent mechanisms of cancer chemoprevention by anti-inflammatory drugs. Front Oncol.

[9] Peterson HI (1983). Effects of prostaglandin synthesis inhibitors on tumor growth and vascularization Experimental studies in the rat. Invas Metastas.

[10] Peterson HI, Alpsten M, Skolnik G, Karlsson L (1985). Influence of a prostaglandin synthesis inhibitor and of thrombocytopenia on tumor blood flow and tumor vascular permeability Experimental studies in the rat. Anticancer Res.

[11] Hofer M, Hoferová Z, Fedoročko P (2002). Hematopoiesis-stimulating and anti-tumor effects of repeated administration of diclofenac in mice with transplanted fibrosarcoma cells. Physiol Res.

[12] Hoferová Z, Fedorocko P, Hofmanová J (2002). The effect of nonsteroidal antiinflammatory drugs ibuprofen, flurbiprofen, and diclofenac on in vitro and in vivo growth of mouse fibrosarcoma. Cancer Invest.

[13] Hixson LJ, Alberts DS, Krutzsch M (1994). Antiproliferative effect of nonsteroidal antiinflammatory drugs against human colon cancer cells. Cancer Epidemiol Biomarkers Prev.

[14] Seed MP, Brown JR, Freemantle CN (1997). The inhibition of colon-26 adenocarcinoma development and angiogenesis by topical diclofenac in 2.5% hyaluronan. Cancer Res.

[15] Falkowski M, Skogstad S, Shahzidi S (2003). The effect of cyclooxygenase inhibitor diclofenac on experimental murine colon carcinoma. Anticancer Res.

[16] Edrei Y, Gross E, Corchia N (2012). Improved efficacy of a novel anti-angiogenic drug combination (TL-118) against colorectal-cancer liver metastases; MRI monitoring in mice. Br J Cancer.

[17] Johnsen JI, Lindskog M, Ponthan F (2004). Cyclooxygenase-2 is expressed in neuroblastoma, and nonsteroidal anti-inflammatory drugs induce apoptosis and inhibit tumor growth in vivo. Cancer Res.

[18] Johnsen JI, Lindskog M, Ponthan F (2005). NSAIDs in neuroblastoma therapy. Cancer Lett.

[19] Larsson K, Kock A, Idborg H (2015). COX/mPGES-1/PGE_2_ pathway depicts an inflammatory-dependent high-risk neuroblastoma subset. Proc Natl Acad Sci USA.

[20] Komar-Stossel C, Gross E, Dery E (2014). TL-118 and gemcitabine drug combination display therapeutic efficacy in a MYCN amplified orthotopic neuroblastoma murine model – evaluation by MRI. PLoS One.

[21] Zerbini LF, Czibere A, Wang Y (2006). A novel pathway involving melanoma differentiation associated gene-7/interleukin-24 mediates nonsteroidal anti-inflammatory drug-induced apoptosis and growth arrest of cancer cells. Cancer Res.

[22] Zerbini LF, Tamura RE, Correa RG (2011). Combinatorial effect of non-steroidal anti-inflammatory drugs and NF-κB inhibitors in ovarian cancer therapy. PLoS One.

[23] Valle BL, D’Souza T, Becker KG (2013). Non-steroidal anti-inflammatory drugs decrease E2F1 expression and inhibit cell growth in ovarian cancer cells. PLoS One.

[24] Mayorek N, Naftali-Shani N, Grunewald M (2010). Diclofenac inhibits tumor growth in a murine model of pancreatic cancer by modulation of VEGF levels and arginase activity. PLoS One.

[25] Chirasani SR, Leukel P, Gottfried E (2013). Diclofenac inhibits lactate formation and efficiently counteracts local immune suppression in a murine glioma model. Int J Cancer.

[26] Leidgens V, Seliger C, Jachnik B (2015). Ibuprofen and diclofenac restrict migration and proliferation of human glioma cells by distinct molecular mechanisms. PLoS One.

[27] Roller D, Axelrod M, Capaldo B (2012). Synthetic lethal screening with small molecule inhibitors provides a pathway to rational combination therapies for melanoma. Mol Cancer Ther.

[28] Yagi K, Kawasaki Y, Nakamura H (2014). Differential combined effect of COX inhibitors on cell survival suppressed by sorafenib in the HepG2 cell line. Biol Pharm Bull.

[29] Gottfried E, Lang SA, Renner K (2013). New aspects of an old drug—diclofenac targets MYC and glucose metabolism in tumor cells. PLoS One.

[30] Inoue T, Anai S, Onishi S (2013). Inhibition of COX-2 expression by topical diclofenac enhanced radiation sensitivity via enhancement of TRAIL in human prostate adenocarcinoma xenograft model. BMC Urol.

[31] Forget P, Bentin C, Machiels JP (2014). Intraoperative use of ketorolac or diclofenac is associated with improved disease-free survival and overall survival in conservative breast cancer surgery. Brit J Anaesth.

[32] Forget P, Berlière M, van Maanen A (2013). Perioperative ketorolac in high risk breast cancer patients Rationale, feasibility and methodology of a prospective randomized placebo-controlled trial. Med Hypotheses.

[33] Forget P, Machiels J-P, Coulie PG (2013). Neutrophil:lymphocyte ratio and intraoperative use of ketorolac or diclofenac are prognostic factors in different cohorts of patients undergoing breast, lung, and kidney cancer surgery. Ann Surg Oncol.

[34] Breuer S, Maimon O, Appelbaum L (2013). TL-118-anti-angiogenic treatment in pancreatic cancer: a case report. Med Oncol.

[35] Lackner H, Urban C, Kerbl R (1997). Noncytotoxic drug therapy in children with unresectable desmoid tumors. Cancer.

[36] Lackner H, Urban C, Benesch M (2004). Multimodal treatment of children with unresectable or recurrent desmoid tumors: an 11-year longitudinal observational study. J Pediatr Hematol Oncol.

[37] Teshima M, Iwae S, Hirayama Y (2012). Nonsteroidal anti-inflammatory drug treatment for desmoid tumor recurrence after surgery. Otolaryngol Head Neck Surg.

[38] Tao YL, Wang ZJ, Han JG (2012). Inflammatory myofibroblastic tumor successfully treated with chemotherapy and nonsteroidals: a case report. World J Gastroenterol.

[39] Nakanishi M, Rosenberg DW (2013). Multifaceted roles of PGE_2_ in inflammation and cancer. Semin Immunopathol.

[40] Dubois RN (2014). Role of inflammation and inflammatory mediators in colorectal cancer. Trans Am Clin Climatol Assoc.

[41] Giuliano F, Warner TD (1999). Ex vivo assay to determine the cyclooxygenase selectivity of non-steroidal anti-inflammatory drugs. Brit J Pharmacol.

[42] Rowlinson SW, Kiefer JR, Prusakiewicz JJ (2003). A novel mechanism of cyclooxygenase-2 inhibition involving interactions with Ser-530 and Tyr-385. J Biol Chem.

[43] Plescia OJ, Smith AH, Grinwich K (1975). Subversion of immune system by tumor cells and role of prostaglandins. Proc Natl Acad Sci USA.

[44] Lynch NR, Castes M, Astoin M (1978). Mechanism of inhibition of tumour growth by aspirin and indomethacin. Br J Cancer.

[45] Ben-Av P, Crofford LJ, Wilder RL (1995). Induction of vascular endothelial growth factor expression in synovial fibroblasts by prostaglandin E and interleukin-1: a potential mechanism for inflammatory angiogenesis. FEBS Lett.

[46] Amano H, Hayashi I, Endo H (2003). Host prostaglandin E(2)-EP3 signaling regulates tumor-associated angiogenesis and tumor growth. J Exp Med.

[47] Howe LR, Subbaramaiah K, Kent CV (2013). Genetic deletion of microsomal prostaglandin e synthase-1 suppresses mouse mammary tumor growth and angiogenesis. Prostaglandins Other Lipid Mediat.

[48] Von Rahden BHA, Stein HJ, Pühringer F (2005). Coexpression of cyclooxygenases (COX-1, COX-2) and vascular endothelial growth factors (VEGF-A, VEGF-C) in esophageal adenocarcinoma. Cancer Res.

[49] Kaur J, Sanyal SN (2011). Diclofenac, a selective COX-2 inhibitor, inhibits DMH-induced colon tumorigenesis through suppression of MCP-1, MIP-1α and VEGF. Mol Carcinog.

[50] Guo F, Wang Y, Liu J (2015). CXCL12/CXCR4: a symbiotic bridge linking cancer cells and their stromal neighbors in oncogenic communication networks. Oncogene.

[51] Salcedo R, Zhang X, Young HA (2003). Angiogenic effects of prostaglandin E2 are mediated by up-regulation of CXCR4 on human microvascular endothelial cells. Blood.

[52] Colleselli D, Bijuklic K, Mosheimer BA (2006). Inhibition of cyclooxygenase (COX)-2 affects endothelial progenitor cell proliferation. Exp Cell Res.

[53] Kalinski P (2012). Regulation of immune responses by prostaglandin E2. J immunol.

[54] Obermajer N, Muthuswamy R, Odunsi K (2011). PGE 2-induced CXCL 12 production and CXCR4 expression controls the accumulation of human MDSCs in ovarian cancer environment. Cancer Res.

[55] Johnson SD, De Costa AMA, Young MRI (2014). Effect of the premalignant and tumor microenvironment on immune cell cytokine production in head and neck cancer. Cancer.

[56] Baratelli F, Lee JM, Hazra S (2010). PGE (2) contributes to TGF-beta induced T regulatory cell function in human non-small cell lung cancer. Am J Transl Res.

[57] Wasserman J, Blomgren H, Rotstein S (1989). Immunosuppression in irradiated breast cancer patients: in vitro effect of cyclooxygenase inhibitors. B New York Acad Med.

[58] Blomgren H, Rotstein S, Wasserman J (1990). In vitro capacity of various cyclooxygenase inhibitors to revert immune suppression caused by radiation-therapy for breast-cancer. Radiother Oncol.

[59] Sinha P, Clements VK, Fulton AM (2007). Prostaglandin E2 promotes tumor progression by inducing myeloid-derived suppressor cells. Cancer Res.

[60] Ostrand-Rosenberg S, Sinha P (2009). Myeloid-derived suppressor cells: linking inflammation and cancer. J Immunol.

[61] Fujita M, Kohanbash G, Fellows-Mayle W (2011). COX-2 blockade suppresses gliomagenesis by inhibiting myeloid-derived suppressor cells. Cancer Res.

[62] Veltman JD, Lambers MEH, van Nimwegen M (2010). COX-2 inhibition improves immunotherapy and is associated with decreased numbers of myeloid-derived suppressor cells in mesothelioma Celecoxib influences MDSC function. BMC Cancer.

[63] Mao Y, Sarhan D, Steven A (2014). Inhibition of tumor-derived prostaglandin-E2 blocks the induction of myeloid-derived suppressor cells and recovers natural killer cell activity. Clin Cancer Res.

[64] Van Hecken A, Schwartz JI, Depré M (2000). Comparative inhibitory activity of rofecoxib, meloxicam, diclofenac, ibuprofen, and naproxen on COX-2 versus COX-1 in healthy volunteers. J Clin Pharmacol.

[65] Schwartz JI, Dallob AL, Larson PJ (2008). Comparative inhibitory activity of etoricoxib, celecoxib, and diclofenac on COX-2 versus COX-1 in healthy subjects. J Clin Pharmacol.

[66] Ha TY (2009). The role of regulatory T cells in cancer. Immune Netw.

[67] Byrne WL, Mills KHG, Lederer JA (2011). Targeting regulatory T cells in cancer. Cancer Res.

[68] Darrasse-Jèze G, Podsypanina K (2013). How numbers, nature, and immune status of Foxp3+ regulatory T-cells shape the early immunological events in tumor development. Front Immunol.

[69] Baratelli F, Lin Y, Zhu L (2005). Prostaglandin E2 induces FOXP3 gene expression and T regulatory cell function in human CD4+ T cells. J Immunol.

[70] Bergmann C, Strauss L, Zeidler R (2007). Expansion of human T regulatory type 1 cells in the microenvironment of cyclooxygenase 2 overexpressing head and neck squamous cell carcinoma. Cancer Res.

[71] Yaqub S, Henjum K, Mahic M (2008). Regulatory T cells in colorectal cancer patients suppress anti-tumor immune activity in a COX-2 dependent manner. Cancer Immunol Immunother.

[72] Lee SY, Choi HK, Lee KJ (2009). The immune tolerance of cancer is mediated by IDO that is inhibited by COX-2 inhibitors through regulatory T cells. J immunother.

[73] Lönnroth C, Andersson M, Arvidsson A (2008). Preoperative treatment with a non-steroidal anti-inflammatory drug (NSAID) increases tumor tissue infiltration of seemingly activated immune cells in colorectal cancer. Cancer Immun.

[74] Ogawa F, Amano H, Eshima K (2014). Prostanoid induces premetastatic niche in regional lymph nodes. J Clin invest.

[75] Sombroek CC, Stam AGM, Masterson AJ (2002). Prostanoids play a major role in the primary tumor-induced inhibition of dendritic cell differentiation. J Immunol.

[76] Shiraishi H, Yoshida H, Saeki K (2008). Prostaglandin E2 is a major soluble factor produced by stromal cells for preventing inflammatory cytokine production from dendritic cells. Int Immunol.

[77] Eruslanov E, Daurkin I, Ortiz J (2010). Pivotal advance: tumor-mediated induction of myeloid-derived suppressor cells and M2-polarized macrophages by altering intracellular PGE_2_ catabolism in myeloid cells. J Leukoc Biol.

[78] Krause P, Bruckner M, Uermösi C (2009). Prostaglandin e2 enhances T-cell proliferation by inducing the costimulatory molecules OX40L, CD70, and 4-1BBL on dendritic cells. Blood.

[79] Yen JH, Khayrullina T, Ganea D (2008). PGE_2_-induced metalloproteinase-9 is essential for dendritic cell migration. Blood.

[80] De Keijzer S, Meddens MBM, Torensma R (2013). The multiple faces of prostaglandin E2 G-protein coupled receptor signaling during the dendritic cell life cycle. Int J Mol Sci.

[81] Trabanelli S, Lecciso M, Salvestrini V (2015). PGE_2_-induced IDO1 inhibits the capacity of fully mature DCs to elicit an in vitro antileukemic immune response. J Immunol Res.

[82] Kusuhara H, Matsuyuki H, Matsuura M (1998). Induction of apoptotic DNA fragmentation by nonsteroidal anti-inflammatory drugs in cultured rat gastric mucosal cells. Eur J Pharmacol.

[83] Kusuhara H, Komatsu H, Sumichika H (1999). Reactive oxygen species are involved in the apoptosis induced by nonsteroidal anti-inflammatory drugs in cultured gastric cells. Eur J Pharmacol.

[84] Shiff SJ, Qiao L, Tsai LL (1995). Sulindac sulfide, an aspirin-like compound, inhibits proliferation, causes cell cycle quiescence, and induces apoptosis in HT-29 colon adenocarcinoma cells. J Clin Invest.

[85] Elder DJE, Hague A, Hicks DJ (1996). Differential growth inhibition by the aspirin metabolite salicylate in human colorectal tumor cell lines: enhanced apoptosis in carcinoma and in vitro-transformed adenoma relative to adenoma cell lines. Cancer Res.

[86] Sawaoka H, Kawano S, Tsuji S (1998). Cyclooxygenase-2 inhibitors suppress the growth of gastric cancer xenografts via induction of apoptosis in nude mice. Am J Physiol.

[87] Ashton M, Hanson PJ (2002). Disparate effects of non-steroidal anti-inflammatory drugs on apoptosis in guinea-pig gastric mucous cells: inhibition of basal apoptosis by diclofenac. Brit J Pharmacol.

[88] Gardner SH, Hawcroft G, Hull MA (2004). Effect of nonsteroidal anti-inflammatory drugs on beta-catenin protein levels and catenin-related transcription in human colorectal cancer cells. Brit J Cancer.

[89] Lu D, Cottam HB, Corr M (2005). Repression of beta-catenin function in malignant cells by nonsteroidal antiinflammatory drugs. P Natl Acad Sci USA.

[90] Inoue A, Muranaka S, Fujita H (2004). Molecular mechanism of diclofenac-induced apoptosis of promyelocytic leukemia: dependency on reactive oxygen species, Akt, Bid, cytochrome c, and caspase pathway. Free Radical Bio Med.

[91] Rana C, Piplani H, Vaish V (2015). Downregulation of PI3-K/Akt/PTEN pathway and activation of mitochondrial intrinsic apoptosis by diclofenac and curcumin in colon cancer. Mol Cell Biochem.

[92] Albano F, Arcucci A, Granato G (2013). Markers of mitochondrial dysfunction during the diclofenac-induced apoptosis in melanoma cell lines. Biochimie.

[93] Singh R, Cadeddu RP, Fröbel J (2011). The non-steroidal anti-inflammatory drugs sulindac sulfide and diclofenac induce apoptosis and differentiation in human acute myeloid leukemia cells through an AP-1 dependent pathway. Apoptosis.

[94] Braun FK, Al-Yacoub N, Plötz M (2012). Nonsteroidal anti-inflammatory drugs induce apoptosis in cutaneous T-cell lymphoma cells and enhance their sensitivity for TNF-related apoptosis-inducing ligand. J Investig Dermatol.

[95] Braun FK, Fecker LF, Schwarz C (2007). Blockade of death receptor-mediated pathways early in the signaling cascade coincides with distinct apoptosis resistance in cutaneous T-cell lymphoma cells. J Investig Dermatol.

[96] Kopp KLM, Dabelsteen S, Krejsgaard T (2010). COX-2 is a novel target in therapy of mycosis fungoides. Leukemia.

[97] Wang X, Baek SJ, Eling TE (2013). The diverse roles of nonsteroidal anti-inflammatory drug activated gene (NAG-1/GDF15) in cancer. Biochem Pharmacol.

[98] Okazaki R, Moon Y, Norimura T (2006). Ionizing radiation enhances the expression of the nonsteroidal anti-inflammatory drug-activated gene (NAG1) by increasing the expression of TP53 in human colon cancer cells. Radiat Res.

[99] Kim CH, Kim MY, Moon JY (2008). Implication of NAG-1 in synergistic induction of apoptosis by combined treatment of sodium salicylate and PI3K/MEK1/2 inhibitors in A549 human lung adenocarcinoma cells. Biochem Pharmacol.

[100] Wang C, Wang J, Bai P (2011). Troglitazone induces apoptosis in gastric cancer cells through the NAG-1 pathway. Mol Med Rep.

[101] Kim KS, Yoon JH, Kim JK (2004). Cyclooxygenase inhibitors induce apoptosis in oral cavity cancer cells by increased expression of nonsteroidal anti-inflammatory drug-activated gene. Biochem Biophys Res Commun.

[102] Gay LJ, Felding-Habermann B (2011). Contribution of platelets to tumour metastasis. Nature Rev Cancer.

[103] Digklia A, Voutsadakis IA (2014). Thrombocytosis as a prognostic marker in stage III and IV serous ovarian cancer. Obstet Gynecol Sci.

[104] Rachidi S, Wallace K, Day TA (2014). Lower circulating platelet counts and antiplatelet therapy independently predict better outcomes in patients with head and neck squamous cell carcinoma. J Hematol Oncol.

[105] Kim KH, Park TY, Lee JY (2014). Prognostic significance of initial platelet counts and fibrinogen level in advanced non-small cell lung cancer. J Korean Med Sci.

[106] Su BB, Chen JH, Shi H (2014). Aspirin may modify tumor microenvironment via antiplatelet effect. Med Hypotheses.

[107] Mikami J, Kurokawa Y, Takahashi T (2015). Antitumor effect of antiplatelet agents in gastric cancer cells: an in vivo and in vitro study. Gastric Cancer.

[108] Mousa SA, Petersen LJ (2009). Anti-cancer properties of low-molecular-weight heparin: preclinical evidence. Thromb Haemost.

[109] Pfankuchen DB, Stölting DP, Schlesinger M (2015). Low molecular weight heparin tinzaparin antagonizes cisplatin resistance of ovarian cancer cells. Biochem Pharmacol.

[110] Bajaj P, Ballary CC, Dongre NA (2004). Comparison of the effects of parecoxib and diclofenac in preemptive analgesia: a prospective, randomized, assessor-blind, single-dose, parallel-group study in patients undergoing elective general surgery. Curr Ther Res Clin Exp.

[111] Palayoor ST, Tofilon PJ, Coleman CN (2003). Ibuprofen-mediated reduction of hypoxia-inducible factors HIF-1alpha and HIF-2alpha in prostate cancer cells. Clin Cancer Res.

[112] Sareddy GR, Kesanakurti D, Kirti PB (2013). Nonsteroidal anti-inflammatory drugs diclofenac and celecoxib attenuates Wnt/β-catenin/Tcf signaling pathway in human glioblastoma cells. Neurochem Res.

[113] Surowiak P, Materna V, Matkowski R (2005). Relationship between the expression of cyclooxygenase 2 and MDR1/P-glycoprotein in invasive breast cancers and their prognostic significance. Breast Cancer Res.

[114] de Groot DJA, de Vries EGE, Groen HJM (2007). Non-steroidal anti-inflammatory drugs to potentiate chemotherapy effects: from lab to clinic. Crit Rev Oncol/Hematol.

[115] Hiľovská L, Jendželovský R, Fedoročko P (2015). Potency of non-steroidal anti-inflammatory drugs in chemotherapy. Mol Clin Oncol.

[116] Legge F, Paglia A, D’Asta M (2011). Phase II study of the combination carboplatin plus celecoxib in heavily pre-treated recurrent ovarian cancer patients. BMC Cancer.

[117] White DL, Dang P, Engler J (2010). Functional activity of the OCT-1 protein is predictive of long-term outcome in patients with chronic-phase chronic myeloid leukemia treated with imatinib. J Clin Oncol.

[118] Wang J, Hughes TP, Kok CH (2012). Contrasting effects of diclofenac and ibuprofen on active imatinib uptake into leukaemic cells. Brit J Cancer.

[119] Kurtova AV, Xiao J, Mo Q (2014). Blocking PGE-induced tumour repopulation abrogates bladder cancer chemoresistance. Nature.

[120] Crokart N, Radermacher K, Jordan BF (2005). Tumor radiosensitization by antiinflammatory drugs: evidence for a new mechanism involving the oxygen effect. Cancer Res.

[121] Forget P, Vandenhende J, Berliere M (2010). Do intraoperative analgesics influence breast cancer recurrence after mastectomy? A retrospective analysis. Anesth Analg.

[122] Retsky M, Demicheli R, Hrushesky WJM (2013). Reduction of breast cancer relapses with perioperative non-steroidal anti-inflammatory drugs: new findings and a review. Curr Med Chem.

[123] Antonio N, Bønnelykke-Behrndtz ML, Ward LC (2015). The wound inflammatory response exacerbates growth of pre-neoplastic cells and progression to cancer. EMBO J.

[124] Neeman E, Ben-Eliyahu S (2013). Surgery and stress promote cancer metastasis: new outlooks on perioperative mediating mechanisms and immune involvement. Brain Behav Immun.

[125] Gottschalk A, Sharma S, Ford J (2010). The role of the perioperative period in recurrence after cancer surgery. Anesth Analg.

[126] Opitz I, Arni S, Oberreiter B (2013). Perioperative diclofenac application during video-assisted thoracic surgery pleurodesis modulates early inflammatory and fibrinolytic processes in an experimental model. Eur Surg Res.

[127] Ash SA, Buggy DJ (2013). Does regional anaesthesia and analgesia or opioid analgesia influence recurrence after primary cancer surgery? An update of available evidence. Best Pract Res Clin Anaesthesiol.

[128] Shakhar G, Ben-Eliyahu S (2003). Potential prophylactic measures against postoperative immunosuppression: could they reduce recurrence rates in oncological patients?. Ann Surg Oncol.

[129] Heaney Á, Buggy DJ (2012). Can anaesthetic and analgesic techniques affect cancer recurrence or metastasis?. Brit J Anaesth.

[130] Horowitz M, Neeman E, Sharon E (2015). Exploiting the critical perioperative period to improve long-term cancer outcomes. Nature Rev Clin Oncol.

[131] Retsky M, Rogers R, Demicheli R (2012). NSAID analgesic ketorolac used perioperatively may suppress early breast cancer relapse: particular relevance to triple negative subgroup. Breast Cancer Res Treat.

[132] Guo Y, Kenney SR, Cook LS (2015). A novel pharmacologic activity of ketorolac for therapeutic benefit in ovarian cancer patients. Clin Cancer Res.

[133] Pantziarka P, Bouche G, Meheus L (2014). Repurposing drugs in oncology (ReDO)-cimetidine as an anti-cancer agent. Ecancermedicalscience.

[134] Deva S, Jameson M (2012). Histamine type 2 receptor antagonists as adjuvant treatment for resected colorectal cancer. Cochrane Database Syst Rev.

[135] Castellone MD, Teramoto H, Williams BO (2005). Prostaglandin E2 promotes colon cancer cell growth through a Gs-axinbeta-catenin signaling axis. Science.

[136] Greenhough A, Smartt HJM, Moore AE (2009). The COX-2/PGE_2_ pathway: key roles in the hallmarks of cancer and adaptation to the tumour microenvironment. Carcinogenesis.

[137] Kaur J, Sanyal SN (2010). PI3-kinase/Wnt association mediates COX-2/PGE_2_ pathway to inhibit apoptosis in early stages of colon carcinogenesis: chemoprevention by diclofenac. Tumor Biol.

